# Extraction Kinetics of Total Polyphenols, Flavonoids, and Condensed Tannins of Lentil Seed Coat: Comparison of Solvent and Extraction Methods

**DOI:** 10.3390/foods10081810

**Published:** 2021-08-05

**Authors:** Fernanda Galgano, Roberta Tolve, Teresa Scarpa, Marisa Carmela Caruso, Luigi Lucini, Biancamaria Senizza, Nicola Condelli

**Affiliations:** 1 School of Agricultural, Forestry, Food and Environmental Sciences (SAFE), University of Basilicata, 85100 Potenza, Italy; fernanda.galgano@unibas.it (F.G.); teresa.scarpa.donofrio@gmail.com (T.S.); marisa.caruso@unibas.it (M.C.C.); nicola.condelli@unibas.it (N.C.); 2 Department of Biotechnology, University of Verona, 37134 Verona, Italy; 3 Department for Sustainable Food Process, Università Cattolica del Sacro Cuore, 29122 Piacenza, Italy; luigi.lucini@unicatt.it (L.L.); biancamaria.senizza@unicatt.it (B.S.)

**Keywords:** by-product, empirical model, extraction kinetic, lentils’ seed coat, phenolic compounds

## Abstract

The lentil seed coat is a waste by-product still rich in phenolic compounds, specifically condensed tannins. The effect of different solvents, as well as different processes, namely conventional solid–liquid extraction (CSLE) and ultrasound-assisted extraction (UAE), on the extraction yield of specific phenolic compound classes was studied. Four empirical two-parameter models were examined to select the one that better fit the experimental data obtained under different operating conditions. Additionally, ultra-high-pressure liquid chromatography coupled to quadrupole-time-of-flight mass spectrometry (UHPLC-ESI/QTOF-MS) was employed to profile the phenolic compounds obtained under distinct extraction conditions. In the operative conditions adopted here, the bioactive compounds yield achieved using UAE was lower than that obtained with CSLE. The kinetics of polyphenols, flavonoids, and condensed tannins extraction from the lentil seed coat were successfully fitted to the power-law models, yielding mean values of the root mean square < 5.4%, standard error of estimation < 0.53, and coefficient of determination > 0.8. In addition, the UHPLC-ESI/QTOF-MS of the lentil seed coat extracts allowed the putative recognition of nearly 500 compounds, mainly flavonoids and phenolic acids.

## 1. Introduction

Pulses are edible seeds and represent an important component of human nutrition in many regions worldwide [1]. As with most pulses, lentils (*Lens culinaris* L.) are an important source of protein and dietary fiber and are rich in micronutrients [2,3]. However, the bioavailability of specific micronutrients, including Fe, might be compromised because of the presence of phytic acids, phenolic compounds, trypsin inhibitors, oxalates, and dietary fiber [3]. Because of this, the demand for decorticated lentils is increasing and the fate of the obtained by-product is typically animal feeding or compost production [4]. However, the lentil seed coat, the obtained by-product, is rich in bioactive molecules. This residue still contains health-promoting bioactive compounds, such as polyphenols, condensed tannin, and flavonoids [5,6]. Several phenolic compounds have been detected in a variety of lentil seed coats, including catechins, procyanidins, quercetin, myricetin, luteolin, apigenin, as well as dimer, trimer, and tetramer proanthocyanidins [7]. As a consequence, the recovery of these high-added-value molecules from agro-industrial wastes has recently become a burning issue [8]. The features of most phenolic compounds range from polar to nonpolar in nature and, because of this, their extraction is often challenging [9]. Typically, a mixture of organic solvents and water is recognized as an advantageous way for the extraction of polyphenolic compounds from plant materials, and, among others, methanol and methanol-water mixtures generally guarantee the highest yield of extraction from plant material [10]. However, methanol is highly toxic and is obtained from natural gas that is a nonrenewable source [11]. Therefore, the selection of more eco-friendly solvents, such as ethanol or water, is of interest. To be capable of recovering a high amount of bioactive molecules from plant material, yielding extraction methods are required. Conventional solid-liquid extraction (CSLE), also named leaching, has extensive applications in the return of a component or mixture of components from plants by dissolving in a proper solvent. Nowadays, more environmentally friendly processes with shorter extraction times and less organic solvent are of great concern. Ultrasound-assisted extraction (UAE) is generally applied as it offers several advantages compared to CSLE, such as solvent consumption reduction, lower energy input, and high reproducibility [4,12]. To select the most suitable extraction conditions, experimental data are frequently utilized for mathematical modeling. Specifically, kinetic models are crucial to understand how complex diffusion and mass may affect the extraction. Empirical kinetic models have been adopted in order to mathematically explain the variation in a bioactive compound concentration over time during the extraction process [13]. Mathematical extraction modeling has already been used to understand, among others, the aqueous extractable tea components [14], the oil extraction from *Terminalia catappa* L. kernel [15], and the extraction of polyphenols from brewer’s spent grain [16], and some of the models applied include the parabolic, power-law, hyperbolic, and Elovich’s [13,17,18,19,20,21] models. Applying the above-mentioned models, it is possible to calculate the related extraction parameters such as the effective diffusion coefficients and extraction rates. To the best our knowledge, these models were never used before to examine the CSLE and UAE of polyphenols compounds from the lentil seed coat. Against that background, this research aimed to compare the yield extraction and determine the proper kinetic model for the different solvent’s CSLE and UAE of phenolic compounds. In addition, to better understand the effect of different methods or solvent on the yield, extract characterization through spectrophotometer assays and ultra-high-performance liquid chromatography-quadrupole time-of-flight (UHPLC-QTOF) mass spectrometry was carried out.

## 2. Materials and Methods

### 2.1. Materials and Methods

#### 2.1.1. Sample Preparation

Lentils of the Laird variety (Lens esculenta Moench) with a flattened lenticular shape and a green color were used. The lentil seed coat was separated from the cotyledon using a semi-industrial husker (OTAKE, model FS20-SM) at a rotor speed of 2300 rpm. The obtained hull was collected, vacuum-packed in plastic bags, and stored until extraction with conventional solid–liquid extraction or ultrasound-assisted extraction. All the solvents used were LC-MS grade, VWR (≥99.9%). 

#### 2.1.2. Conventional Solid–Liquid Extraction (CSLE)

Phenolic compounds were obtained from the lentil seed coat following the method reported by Zhang et al. [10] with slight modification. Briefly, 200 mg of the lentil’s hull was weighed into a 50 mL screw-capped plastic tube covered with aluminum foil and extracted at 170 rpm in an orbital shaker (Thermolyne AROS 160, Barnstead International, Boston, MA, USA) at room temperature using the appropriate solvent. Specifically, three different solutions were utilized: (a) MeOH: H_2_O (70:30) (*v/v*), (b) EtOH:H_2_O (60:40) (*v/v*), and (c) deionized water. As the increased acidity supports the dissolution of phenolic compounds [22], all the extraction solvents were acidified with 0.1% HCl (*v/v*). After 15 h of extraction, the mixture was centrifuged at 3000× *g* for 10 min. After collecting the supernatant, the extracted lentil seed coat was re-extracted twice more, using each time 10 mL of the same solvent and following the same conditions. A suitable quantity of the fresh solvent was then added to the collected supernatants, bringing the final volume to 40 mL. Lastly, before the spectrophotometric assays, the extracts were filtered through a 0.45 µm PTFE membrane filter.

#### 2.1.3. Ultrasound-Assisted Extraction (UAE) 

Ultrasound-assisted extraction with a 250 W rated output power and 40 kHz frequency was conducted on 200 mg of lentil seed coats in 40 mL of solvents at room temperature. The maximum extraction time was 2 h, based on the preliminary data obtained. Two different solvents were used: EtOH:H_2_O (60:40) (*v/v*) and deionized water, both acidified with 0.1% HCl (*v/v*). After the extraction, the mixture was centrifuged at 3000× *g* for 10 min. A suitable quantity of the fresh solvent was then added to the collected supernatants, bringing the final volume to 40 mL. Lastly, before the spectrophotometric assays, the extracts were filtered through a 0.45 µm PTFE membrane filter.

#### 2.1.4. Total Phenolic Compounds (TPC) Determination

The total phenolic compounds in the lentil seed coats extracted as above were evaluated, as described by Singleton and Rossi [23]. In detail, 100 μL of the extract was mixed with 500 μL of 10-fold-diluted Folin-Ciocalteau reagent at room temperature. After 10 min, 500 μL of a saturated solution of Na_2_CO_3_ was added. The reaction mixture was incubated at room temperature for 30 min. The absorbance was determined at 765 nm (Cary 1E UV–VIS spectrophotometer, Varian, Agilent, Milano, Italy). The TPC was expressed as milligrams of gallic acid equivalents (GAE) per gram of dry weight (dw).

#### 2.1.5. The Total Flavonoid Content (TFC) Determination

The total flavonoid content (TFC) assay was performed, as reported by Dewanto et al. [24]. In detail, 100 μL of the extraction samples was mixed with 440 μL of 0.066 M of NaNO_2_ solution at room temperature. After 5 min, 60 μL of AlCl_3_ 0.75 M was mixed and allowed to react for another 6 min before the addition of 400 μL of NaOH 0.5 M. The absorbance was determined at 500 nm using a spectrophotometer. The results were expressed as milligrams of catechin equivalents (CAE) per gram of dry weight (dw).

#### 2.1.6. The Condensed Tannin Content (CTC) Determination

The condensed tannin content (CTC) of the extracted samples was performed by the vanillin assay [25]. Eight hundred microliters of vanillin reagent was added to 200 μL of the extract. Samples were incubated for 20 min at room temperature and, finally, the absorbance was recorded at 500 nm. The results were reported as milligrams of catechin equivalents (CAE) per gram of dry weight (dw).

#### 2.1.7. Kinetic Modeling for the Extraction Methods 

To evaluate the entire extraction process, four experimental kinetic models, generally implemented in modeling the extraction of solutes from solid materials, were employed to fit the experimental data, namely the parabolic diffusion, power-law, hyperbolic, and Elovich’s models. 

When applied to the extraction of plant materials, the parabolic diffusion equation can be written (Equation (1)) as:

(1) $$\bar{q}=A_{0}+A_{1}t^{1/2}$$

This model agrees with the two-step extraction process consisting of the washing of weakly bound material, which is instantaneously leached, followed by the diffusive release. In this sense, the parabolic diffusion model parameter A_0_ is the washing coefficient instead, and A_1_ represents the diffusion rate constant (min^−0.5^).

The power-law model explains the extraction mechanism by the diffusion of solute through a nonswelling material (Equation (2)):

(2) $$\bar{q}=Bt^{n}$$

where n and B are the diffusional exponent and a constant of the model, respectively. When the extraction is made from plant materials, n < 1. The hyperbolic model, also known as Peleg’s model, can be written as Equation (3):

(3) $$\bar{q}=\frac{C_{1}t}{1+{\;C}_{2}t}$$

where C_1_ is the extraction rate at the beginning (min^−1^) and C_2_ is the constant related to the maximum extraction yield (min^−1^).

Elovich’s model (Equation (4)) refers to a logarithmic relation:

(4) $$\bar{q}=E_{0}+E_{1}\;\ln t$$

where E_0_ and E_1_ are parameters of the Elovich equation. 

The nonlinear kinetic equations and the linearized form of these models are presented in Table 1. 

The reported models were based on the following general assumptions:

- All particles were sphere-shaped with a uniform size.
- The solute component was uniformly distributed in the matrix.
- The diffusion coefficient of the solute components was constant.
- Solid particles were well distributed in the extracting solvent.

The TPC, TFC, and CTC yields of the lentil seed coat were calculated according to the following formula (Equation (5)):

(5) $$\mathrm{Yield}\%=\frac{weight\;of\;bioactive\;compound\;extracted\;\left(g\right)}{weight\;of\;lentils\;seed\;coat\;\left(g\right)}\;\times \;100\;$$

#### 2.1.8. Characterization of the Extracts by UHPLC-ESI/QTOF Mass Spectrometry 

The phytochemical profile of the different extracted matrix was investigated through an UHPLC-ESI/QTOF-MS, as previously reported by Rocchetti et al. [26]. Briefly, the chromatographic separation used, as mobile phases, a mixture of acetonitrile and water (LC-MS grade, VWR, Milan, Italy), both acidified with 0.1% formic acid (*v/v*), and a C18 column Agilent Zorbax eclipse plus (50 mm × 2.1 mm, 1.8 μm). The acquisition of accurate masses was made at 30.000 FWHM in a positive full scan (100–1200 m/z range). The injection volume was 6 μL, and source conditions were as follows: nitrogen operated as a drying gas (8 L/min and 330 °C) and sheath gas (10 L/min and 350 °C), the capillary voltage was 3.5 kV, and the nozzle voltage was 300 V.

The Profinder B.06 software (from Agilent Technologies) was used for compound annotation from raw mass features, in accordance with the algorithm ‘find-by-formula.’ For annotation purposes, after post-acquisition filters, the entire isotope pattern (i.e., monoisotopic mass, isotopic spacing, and isotopic ratio) and a 5 ppm tolerance for mass accuracy were used to achieve a level 2 of confidence (i.e., putatively annotated compounds) of the Metabolomics Standard Initiative [27]. The database employed for annotations was Phenol-Explorer 3.6 (www.phenol-explorer.eu, accessed 26 May 2021), and the compounds considered and included in the dataset were those identified within 100% of replications in at least one treatment.

Afterward, the compounds annotated were classified according to their respective phenolic class/subclass, and the cumulative abundances were determined from calibration curves of pure standard solutions (Extrasynthese, Lyon, France; purity > 98%). To this aim, cyanidin, catechin, quercetin, luteolin (flavonoids), ferulic acid (phenolic acids), tyrosol (low-molecular-weight compounds), sesamin (lignans), and resveratrol (stilbenes) were used for the quantification as the representative phenolic compounds, and the results were expressed as mg equivalents/kg dry weight (dw).

### 2.2. Statistical Analysis

All data represent the means of at least three measurements. The means’ assessment was conducted using the ANOVA with a post hoc Tukey test at *p* < 0.05. Statistical analyses were performed by XLSTAT (Addinsoft SARL, Paris, France). The Solver Microsoft Excel package (Microsoft Corporation, Redmond, WA, USA) was used to evaluate data fittings using nonlinear regression. To assess the level at which models interpret the experimental data, the coefficient of determination (R^2^), root-mean-square (RMS), and standard error of estimation (SEE) were, respectively, calculated using the following equations:

(6) $$R^{2}=1-\frac{\sum_{N=1}^{N}{\left({\bar{q}}_{\exp}-{\bar{q}}_{\mathrm{cal}}\right)}^{2}}{\sum_{N=1}^{N}{\left({\bar{q}}_{\exp}-{\bar{q}}_{\mathrm{cal}}\right)}^{2}}$$

(7) $$\mathrm{RMS}={\sqrt{\frac{1}{N}\sum_{i=1}^{N}\left(\frac{{\bar{q}}_{\exp}-{\bar{q}}_{\mathrm{cal}}}{{\bar{q}}_{\exp}}\right)}}^{2}$$

(8) $$\mathrm{SEE}=\sqrt{\sum^{\;}{\frac{\left(x-y\right)}{dt}}^{2}}$$

where *N* is the number of experimental data points. $\bar{q}$_cal_ and $\bar{q}$_exp_ are the estimated and experimental values, respectively, in Equations (2) and (3), while *x* and *y* are the experimental and calculated values in Equation (4). *dt* is the change in time. The higher the R^2^ and the lower the RMS and SEE values, the better the goodness of fit.

## 3. Results and Discussion

### 3.1. Conventional Solid-Liquid Extraction and Ultrasound-Assisted Extraction

**Figure 1:**
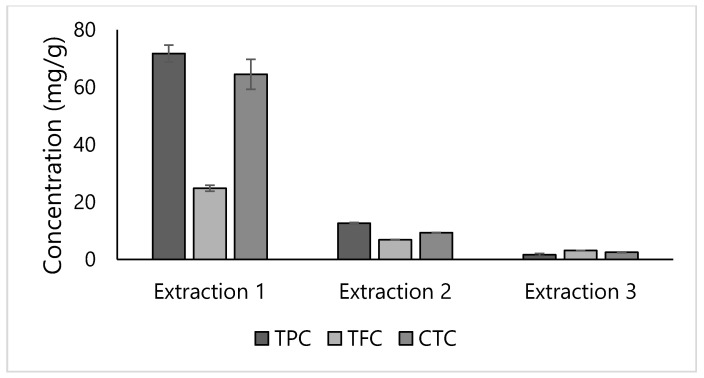
Total phenolic compounds (TPC), total flavonoid compounds (TFC), and total condensed tannins content (CTC) of lentil seed coat obtained using conventional solid–liquid three-time extraction draws of 15 h each with aqueous methanol (MeOH:H_2_O) as solvent.

Flavonoids and condensed tannins, the principal polyphenols in legume seeds, are broadly found in the lentil seed coat [10]. The TPC, TFC, and CTC of the lentil seed coat extracted using a methanol (MeOH:H_2_O) mixture under CSLE conditions are shown in Figure 1. The protocol used here was that reported by Zhang et al. [10], with three-time extraction draws of 15 h each. As it is possible to observe, the three-time extraction of the lentil seed coat, although time-consuming, allowed an extra extraction ranging from 14.48 to 27.72% and from 2.28 to 12.58% for the second and third extractions, respectively, according to the different compounds evaluated. The three-time extraction procedure was more effective, as already reported by Złotek et al. [28], on the basil leaves. Nevertheless, it must be indicated that the extraction time mostly depends on the plant material; a single 60 min extraction allows the higher phenolics recovery from fruit and vegetables matrices [29].

Although the methanolic mixture allowed a higher extraction of phenolic compounds from the lentil seed coat using CSLE, nowadays, more environmentally friendly processes with shorter extraction times and fewer organic solvents are of great concern. Therefore, the possibility to obtain a high yield using ethanol (EtOH:H_2_O) mixture or H_2_O as a solvent, both acidified with HCl 0.1 M, with CSLE and UAE was investigated. 

**Figure 2:**
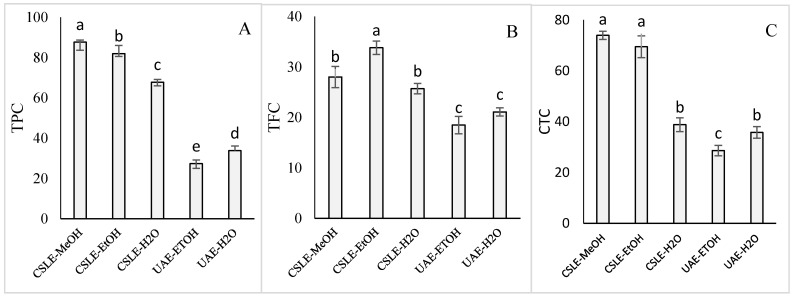
Total phenolic compounds (TPC) (mg/g) (**A**), total flavonoid compounds (TFC) (mg/g) (**B**), and total condensed tannins content (CTC) (mg/g) (**C**) of lentil seed coat extracted by conventional solid-liquid extraction (CSLE) and ultrasound-assisted extraction (UAE) with aqueous methanol (MeOH), aqueous ethanol (EtOH), and water (H_2_O) as extraction solvent. Values with different superscripts within the same graph are significantly different for *p* < 0.05.

The results showed that the use of diverse solvents resulted in different extractions, which is due to the nature and the number of secondary metabolites recovered. In Figure 2, the TPC, TFC, and CTC values obtained using methanol were reported to obtain a facilitated comparison among the different extractions. When CSLE was deployed, the TPC estimation was correlated with the solvent used, based on the relation: methanol  >  ethanol  >  water, with significant differences among the samples. The TPC quantifications were 87.61, 81.94, and 67.82 mg GAE/g of the lentil seed coat (dw) using methanol, ethanol, and water, respectively. These results corroborate with other research in which a mixture of organic solvents and water was allowed to increase the extraction of TPC compared to the use of only water [30,31]. According to Garmus et al. [32], the highest TPC recovery may be ascribed to the kind of solvent and the polarity degree. However, as reported by Naczk and Shahidi [33], the solubility of phenolic compounds is not just dependent on the polarity, but also associated with the degree of polymerization and the bonds with other samples’ ingredients, which may develop insoluble complexes. It should be noted that there is no uniform or fully adequate method for the extraction of all phenolic compounds. Compared to the results reported in the present study, a lower TPC extraction was reported by Zhang et al. [10] in red and green lentils with values ranging from 4.56 to 8.34 mg GAE/g (dw). However, besides considering that different TPC responses could be due to the plant genotype, geographical factor, type of crop, and climatic variable, it must be underlined that in the cited research, the TPC values were evaluated on the whole lentil. TFC values, measured with the aluminum chloride colorimetric assay, were 28.02, 33.80, and 25.70 mg CAE/g of the lentils seed coat (dw) in methanol, ethanol, and water, respectively. Thus, the extraction was according to the relation: ethanol > methanol  =  water, without significant differences between methanol and the water extraction. These results could be ascribed to the solubility of polar carbohydrates and glycosides of secondary metabolites in these solvents, as flavonoid glycosides as their glycoside derivatives are commonly extracted with more polar solvents, such as acetone, methanol, ethanol, and water [34]. Instead, CTC, determined by acidified vanillin assay, was higher in methanolic and ethanolic extracts (73.96 and 64.47 CAE/g of lentil seed coat, respectively) and lower using water (38.82 mg CAE/g of lentil seed coat). As for the TPC, the amounts of TFC and CTC obtained were much higher than those reported by Zhang et al. [10] using the same procedure. The achieved results also indicated that different process extractions (CSLE vs. UAE) resulted in significant variations in the TPC, TFC, and CTC. Specifically, reductions of 49 and 65.7% of the TPC extraction using water and ethanol were detected employing UAE instead of CSLE. Similar trends, albeit less pronounced, were registered for TFC (−17 and 47.7%) and CTC (−5.3 and 58%) when CSLE was substituted with UAE in both aqueous and ethanolic extract. Overall, the yields achieved using UAE were lower compared to the CSLE because it is well known that the ultrasound efficiency may depend on several factors such as sonication amplitude, pulse cycle, extraction time, and the raw materials [35]. Moreover, with regard to the UAE, the different solvents used for the extraction of the lentil seed coat had a significant effect on the TPC and CTC content. The UAE process parameters used here allowed a greater extraction using water compared to the ethanol. These could be attributed to the phenolic compounds’ degradation in the ethanolic extract that could happen over time, as corroborated by the UHPLC-ESI/QTOF mass spectrometry data. As reported elsewhere, the longer the UAE time, the lower the extraction of phenolic compounds observed, and the differences detected in regard to the extraction solvent can be related to the sensitivity of the phenolics, which varies in different types of materials [36]. Meanwhile, some researchers have summarized that a time of sonication higher than 40 min at an energy level higher than 20 kHz could gravely affect the phytochemical extraction yield due to the decreased rate of diffusion area/rate and increased diffusion distance [37,38]. In addition, operating under these conditions may promote the production of involuntary modifications and the assembly of free radicals in the extracted compounds [38]. The TPC was positive with the TFC (0.9013), indicating that the condensed tannins are the most abundant phenolic group in lentil seed coats as previously reported [5,6]. Taken together, our data showed that the solvent selection should be based according to the types of secondary metabolites assumed to be extracted from the lentil seed coat, as the methanolic and the ethanolic extracts had shown the high contents of TPC and CTC, while methanol and water were suitable for the extraction of flavonoids. Furthermore, toxicity, cost, and availability must also be taken into account when solvent selection should be performed [35].

### 3.2. Kinetic Modeling of the Extraction Process 

**Figure 3:**
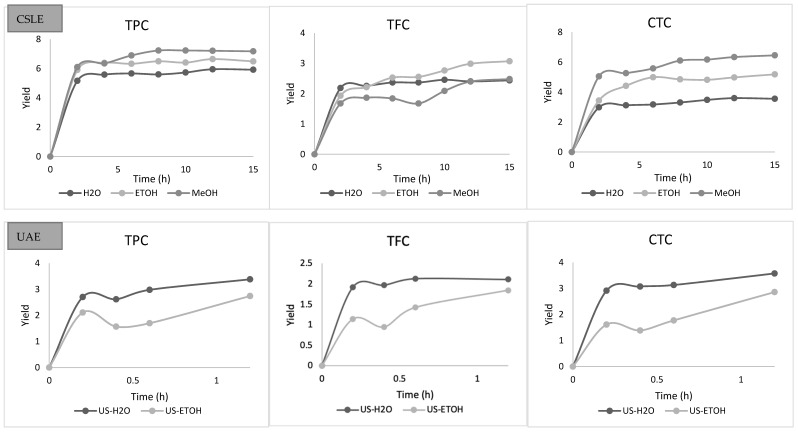
Kinetic yield of total phenolic compounds (TPC), total flavonoid compounds (TFC), and condensed tannin content (CTC) over the extraction time for conventional solid–liquid extraction (CSLE) and ultrasound-assisted extraction (UAE).

**Figure 4:**
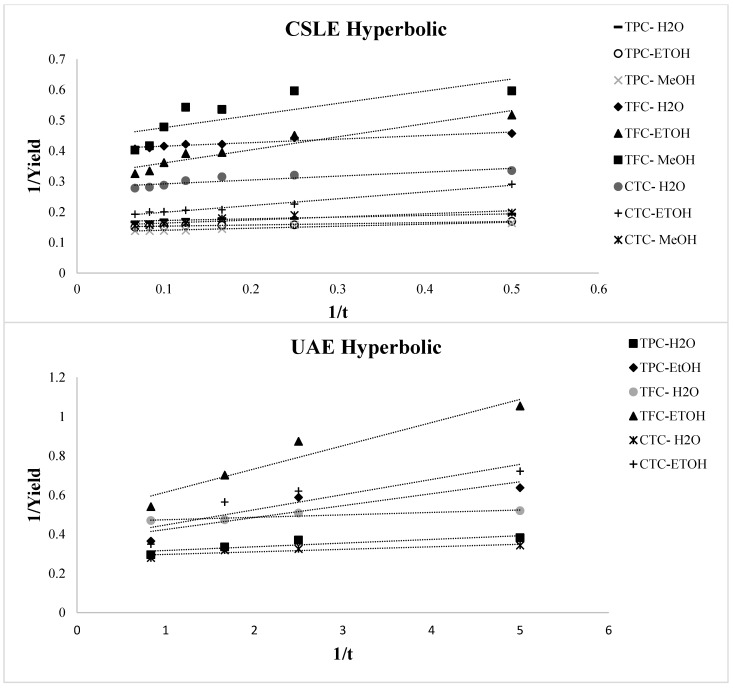
Plot of hyperbolic model for conventional solid-liquid extraction (CSLE) and ultrasound-assisted extraction (UAE) for the total phenolic compound (TPC), total flavonoid compound (TFC), and total condensed tannin compound (CTC) using the different selected solvents.

**Figure 5:**
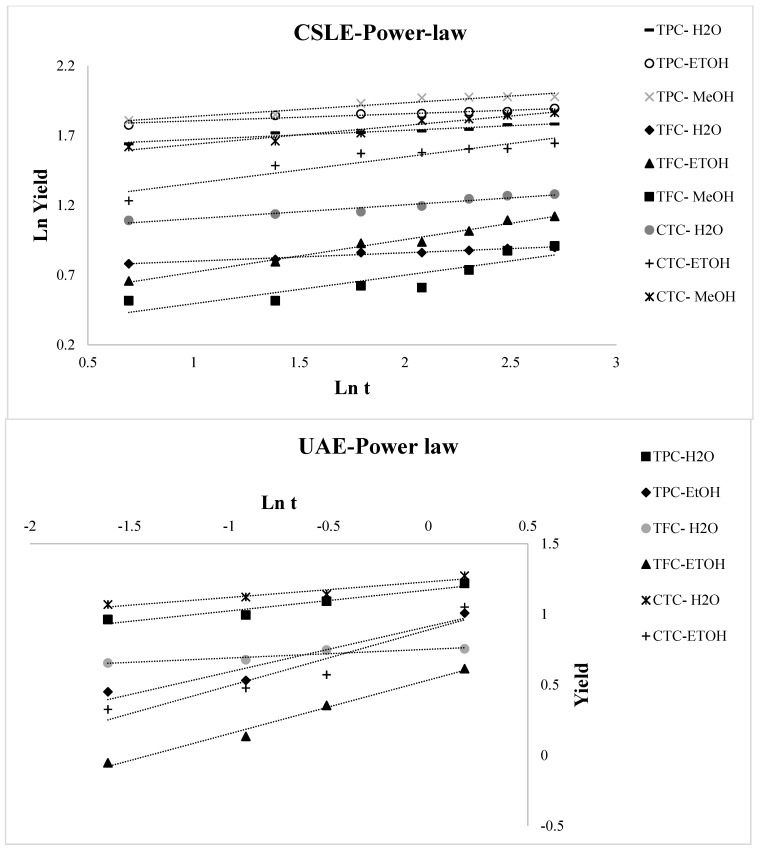
Plot of power-law model for conventional solid-liquid extraction (CSLE) and ultrasound-assisted extraction (UAE) for the total phenolic compound (TPC), total flavonoid compound (TFC), and total condensed tannin compound (CTC) using the different selected solvents.

**Figure 6:**
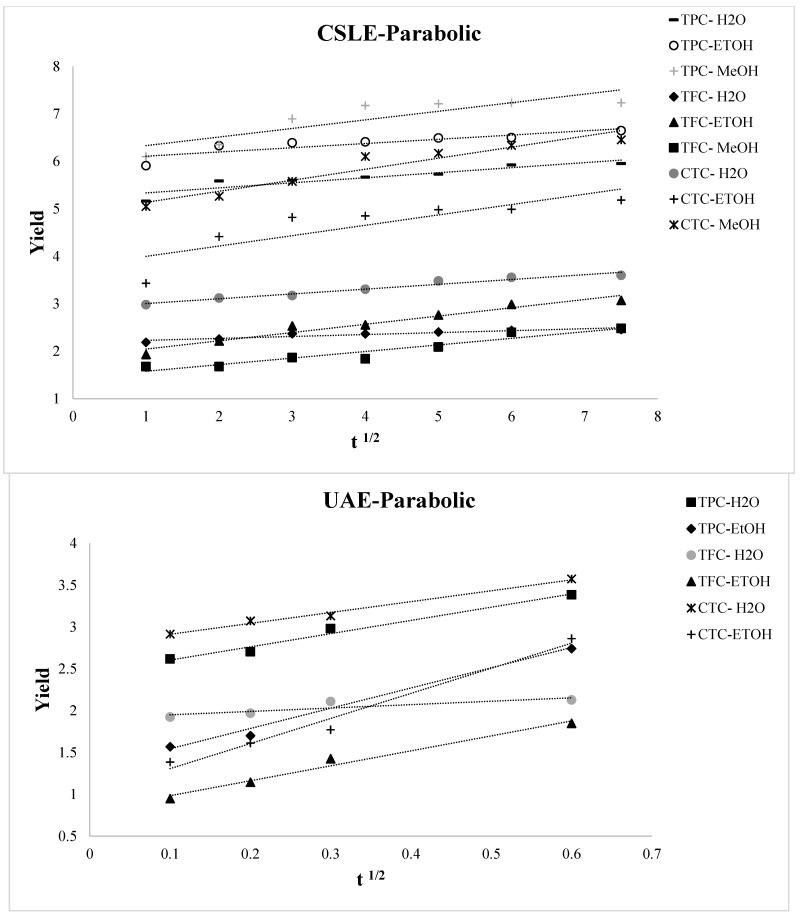
Plot of parabolic model for conventional solid-liquid extraction (CSLE) and ultrasound-assisted extraction (UAE) for the total phenolic compound (TPC), total flavonoid compound (TFC), and total condensed tannin compound (CTC) using the different selected solvents.

**Figure 7:**
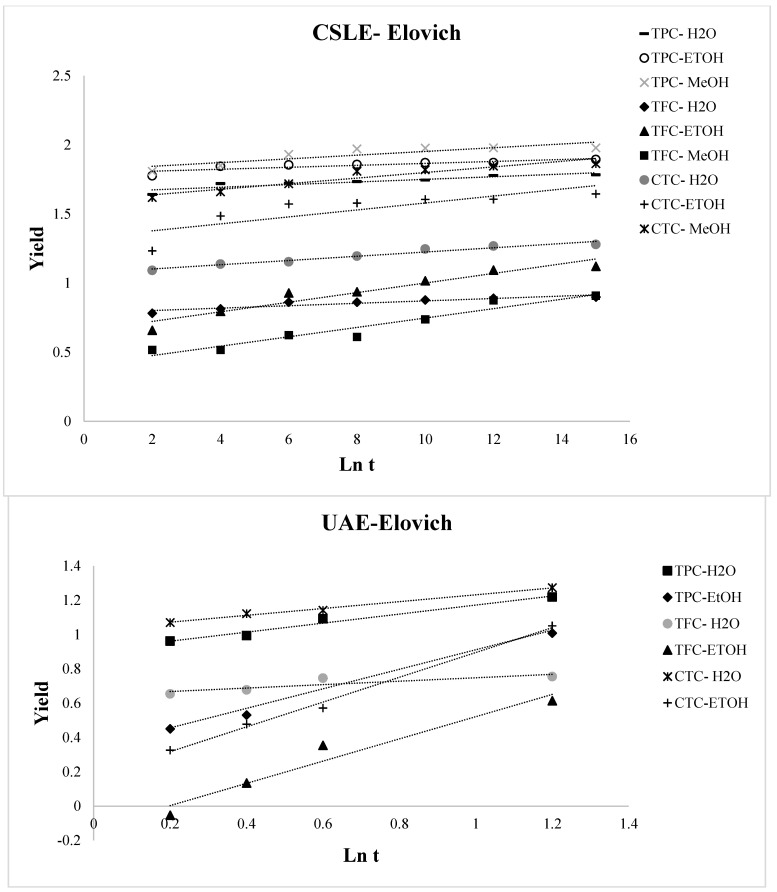
Plot of Elovich’s model for conventional solid-liquid extraction (CSLE) and ultrasound-assisted extraction (UAE) for the total phenolic compound (TPC), total flavonoid compound (TFC), and total condensed tannin compound (CTC) using the different selected solvents.

The variation over time of TPC, TFC, and CTC of the lentil seed coat extracted with different solvents using CSLE and UAE is reported in Figure 3. By analyzing the extraction curves for all the operative conditions selected in this study, two stages of extraction were detected. Specifically, fast increases in the TPC, TFC, and CTC concentrations early in the beginning of the process and gradual increases in the further progress of the extraction process were distinguished. TPC, TFC, and CTC increased quickly with time during the first 120 min for CSLE and 20 min for UAE, then tending to a constant value. For CSLE, we reported here only the data related to the first time of extraction, as the observed trend was similar in the other two extraction’s times. Thus, typical extraction kinetics with a fast step of washing dominated by solute partition first and then a slow step of diffusion controlled by solute diffusion was observed. This is in line with most solid–liquid extraction curves [39,40,41]. Various mathematical simulations can prove this trend, and here, four empirical kinetic models for CSLE and UAE were applied to fit the experimental data for water, and ethanolic and methanolic extractions. The parabolic (Equation (1)), power-law, (Equation (2)), hyperbolic (Equation (3)), and Elovich’s (Equation (4)) equations were fitted to the experimental data and the model parameters were definite (Table 2). Figure 4, Figure 5, Figure 6 and Figure 7 show the plot of extraction yield vs. time for CSLE and UAE and the ultrasound-assisted extraction for TPC, TFC, and CTC using the different selected solvents with the linearized hyperbolic, power-law, parabolic model, and Elovich’s equations.

These models have been previously implemented for the extraction of bioactive compounds from several matrices such as Asteraceae plant, pomegranate peels, barley, and chicory roots by-products [41,42,43,44]. The yield of the phenolic compounds achieved from the nonlinear models was largely close to the experimental one. The criteria used to evaluate the model’s ability to depict the experimental data were the magnitudes of the linear correlation coefficient (R^2^), the SEE and RMS. The greater the value of R^2^ and the lower the value of RMS and SEE, the better the goodness of fit. The statistical R^2^, RMS, and SEE results presented in Table 2 displayed that, regardless of the model applied, the individual values of R^2^ were higher than 0.622, the RMS values were lower than ±10% for all extraction conditions, and SEE was always lower than 1%. Thus, all the evaluated models may potentially be appropriate to understand the extraction of the phenolic compounds from the lentil seed coat using CSLE or UAE with different solvents for engineering purposes [45]. However, as reported by Lafka et al. [46], it must be underlined that because they are empirical models, it is difficult to assign physical meaning to their parameters. It was detected that the RMS and SEE diminished and R^2^ increased in the following order: hyperbolic model → parabolic model → Elovich’s equation → power law model. Figure 4 displays the plot of extraction yield vs. time for CSLE and UAE for TPC, TFC, and CTC using the different selected solvents with the linearized hyperbolic equation. The hyperbolic equation was, among the selected models, the one with the lower fitness degree. The aforementioned models, which depict an extraction kinetic behavior of the first order, with the yield growing linearly with time in the first step and zero order in the very late stage, were not appropriate to describe our data [14]. The parabolic model fitted well the kinetic data of UAE extraction for TPC, TFC, and CTC (except for CSLE of TFC in water), instead of scarcely representing the data obtained by CSLE. Figure 5 shows the plot of the extraction yield vs. time with the parabolic equation. The effect of the extraction process and the solvent was observed on the washing coefficient A_0_ of the parabolic model, which symbolizes that the extraction yield was obtained immediately as the plant material was immersed into the solvent at t = 0. Higher values were obtained in CSLE for all the class compounds analyzed with organic solvents that allowed higher TPC and CTC yields. On the contrary, in UAE extraction, using water, a higher instantaneous TPC, TFC and CTC recovery was obtained. Although Elovich’s model showed a good fit, it did not show a good correlation for the CTC extraction (both in CSLE and UAE) and the CSLE with MeOH (R^2^ = 0.759), and Figure 6 shows the plot of the extraction yield vs. time with Elovich’s equation. The power-law kinetic model best explained the extraction kinetics of TPC, TFC, and CTC from the lentil seed coats using CSLE and UAE, and Figure 7 shows the plot of the extraction yield vs. time with the linearized power-law equation. The best accuracy of the power-law model was assessed based on elevated R^2^ (0.809–1.000) and low RMS (%) (0.394–5.389) and SEE (0.002–0.528), which corroborated the model’s accuracy and suitability to describe the extraction. The power-law is the most relevant model for the extraction of a substance from a nonswelling device [47] with a diffusion exponent n < 1 when the matrix is a plant material [45]. Specifically, in our study, n showed values lower than 0.461, which showed Fickian diffusion-controlled TPC extraction from the lentil seed coats. Analogous results have been presented by Lafka et al. [46] investigating the extraction of phenolic compounds from olive leaves using different extraction solvents with n < 0.5. Lower values of n for TPC, TFC, and CTC were found in both CSLE and UAE for pure water results, in agreement with Kashaninejad et al. [48] who worked on olive leaves. 

### 3.3. Characterization of the Extracts by UHPLC-ESI/QTOF Mass Spectrometry 

An untargeted metabolomic methodology was used to comprehensively evaluate the phenolic composition of the singular lentil extracts, considering both the influence of the extraction solvents and the effect of ultrasound irradiation under different operating conditions.

The whole list of compounds recorded in our experimental conditions is provided as supplementary material (Table S1). This approach has enabled us to putatively annotate 496 compounds, with a considerable abundance of flavonoids (161 compounds) and phenolic acids (65 compounds). Overall, flavones, such as apigenin and luteolin glucosides, and flavonols (i.e., catechin, epicatechin, and gallocatechin) were the most representative compounds in terms of relative abundance. Besides, the class of phenolic acids was characterized by hydroxycinnamics (i.e., ferulic and coumaric acids) and hydroxybenzoics (i.e., 4-hydroxybenzoic and protocatechuic acids). In agreement with our findings, the literature reports that flavonol and flavone glycosides prevail in lentil seed coats, while the cotyledon contains nonflavonoid phenolic compounds, such as hydroxybenzoic and hydroxycinnamic acids [49]. Afterward, the phenolic compounds detected were classified as mg/kg equivalents according to a representative standard per class/sub-class. The results of the semi-quantitative analysis are provided in the supplementary material (Tables S2–S3).

Looking at the results, ethanol was the best solvent promoting the extraction of flavonoids (except for flavonols), phenolic acids, and stilbenes, while methanol-water extracts were particularly abundant in tyrosol equivalents. Indeed, adding water to organic solvents such as methanol and ethanol was found to better promote the extraction of phenolics; using water is not as effective as hydroalcoholic solutions, because polyphenols are broadly soluble in organic solvents with lower polarities than water [50].

With regard to the ultrasound-assisted extraction (UAE), it is known that the efficiency may depend on several factors such as sonication amplitude, pulse cycle, extraction time, and the raw materials [51]. Under our experimental conditions, water extracts showed the highest total phenolic content after 40 min of ultrasound application (3348.56 mg/kg), whilst a lower content was recorded after 20 min of UAE (3048.04 mg/kg). When considering the hydroalcoholic solution, the results showed an opposite trend, as the 20 min of UAE was the most effective technology, counting a total phenolics content of 4656.59 mg/kg, while after 40 min of ultrasound application, the TPC was 3305.81 mg/kg.

In particular, the flavan-3-ols and flavonols were the classes showing the highest recovery after 120 min in both water 100% and aqueous ethanol (60:40) solutions, with increases of 1.2- and 1.3-folds, respectively. Moreover, the most effective extraction of low-molecular-weight compounds was achieved under aqueous conditions after 40 min of UAE (from 278.38 to 318.56 mg/kg). However, considering aqueous ethanol after 20 min of ultrasound application, the total phenolics had a 1.4-fold higher value than that of the control (1621.76 mg/kg vs. 1133.63 mg/kg, respectively).

**Figure 8:**
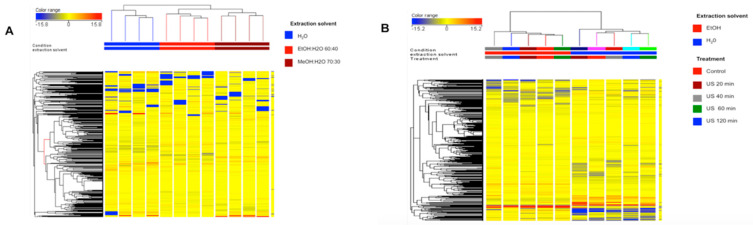
Non-averaged unsupervised cluster analysis on the phenolic profile of different lentil seed coat extractions, as a function of the solvent used (**A**) and solvent-extraction technique used (**B**). The compound’s intensity was used to build up the heat map, on the basis of which the clusters were generated.

**Figure 9:**
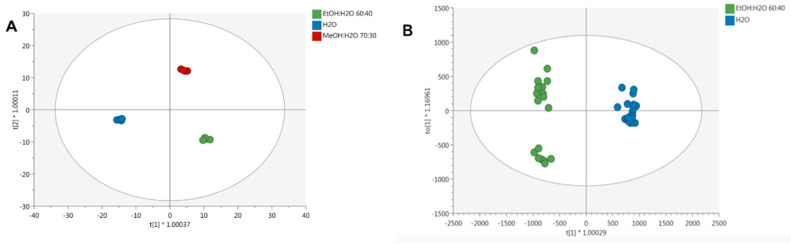
Orthogonal projection to latent structures discriminant analysis (OPLS-DA) on phenolic profiles of different lentil seed coat extracts, as a function of the solvent used (**A**) and solvent-extraction technique used (**B**). Individual replications are given in the class prediction model score plot.

Afterward, unsupervised hierarchical cluster (HCA) analysis was applied in order to naively compare the phenolic profiles under the different extraction solvents tested. As expected, two clusters were generated, with the organic solvents ethanol and methanol demonstrating a comparable phenolic profile, whilst water was separated in a second and more distinct cluster (Figure 8). Interestingly, the second HCA carried out from UAE experiments (Figure 8B) suggested that the extraction solvent used was still hierarchically more important than the UAE time considered. Therefore, a supervised OPLS discriminant analysis was built considering each different extraction condition (Figure 9A). Remarkably, a clear separation between methanolic, ethanolic, and aqueous extracts was achieved, thus supporting what was already outlined by the unsupervised cluster analysis. The model parameters were good, with R2Y (the goodness-of-fit) = 0.99 and Q2Y (goodness-of-prediction) = 0.90; the cross-validation parameters also confirmed the robustness of the OPLS model (CV-ANOVA < 0.01).

Then, the VIP approach (variable importance in projections) was applied to investigate those compounds most influenced by the extraction method used; only those compounds with a VIP score > 1.2 were considered and listed in the supplementary material. The compounds possessing the highest discrimination power (VIP score > 1.4) were flavonoids, in the majority, and flavonols, anthocyanins, and low-molecular-weight compounds (tyrosols). The anthocyanins cyanidin 3-O-glucosyl-rutinoside, cyanidin 3-O-sambubioside 5-O-glucoside, and delphinidin 3-O-rutinoside possessed a VIP score = 1.46. Thereafter, in a second OPLS-DA model (Figure 9B), a clear separation was achieved and pointed out the differences between the ultrasound time (20, 40, 60, and 120 min) application in both aqueous and hydroalcoholic solvents. The model parameters were good, with R2Y (the goodness-of-fit) = 0.98 and Q2Y (goodness-of-prediction) = 0.97. In addition, 38 compounds possessing a VIP score > 1.2 were selected and included flavonoids (mainly anthocyanins) and low-molecular-weight phenolics (alkylphenols). The most discriminant compounds highlighted by the VIP selection method were resorcinols (5-nonadecylresorcinol, VIP = 7.17; 5-heptadecylresorcinol, VIP = 6.96), and cyanidin 3-O-rutinoside and cyanidin 3-O-(6′′-p-coumaroyl-glucoside) with VIP scores of 3.89 and 3.28, respectively. In addition, the hydroxycinnamic acid 3,5-diferuloylquinic acid was found to record a VIP score of 2.39.

## 4. Conclusions

Conventional solid-liquid extraction (CSLE) and ultrasound-assisted extraction (UAE) of the phenolic compounds (specifically total phenolic compounds, total flavonoids, and total condensed tannin content) from the lentil seed coats were studied. Under the operative conditions adopted here, the bioactive compounds yield achieved using UAE were lower compared to the CSLE. The modeling studies on the kinetics of CSLE and UEA on lentil seed coat phenolic compounds displayed that the kinetics was highly conditioned from the process used, as well as on the extraction solvent. All the selected kinetic models fitted the experimental data quite well. However, the power-law model allowed us to obtain lower RMS and SEE and a higher R^2^. Looking at the results, ethanol was the best solvent promoting the extraction of flavonoids, phenolic acids, and stilbenes, while methanol-water extracts were particularly abundant in tyrosol equivalents. Although the water was not as effective as hydroalcoholic solutions, it must be remembered that the choice of solvent should be based not only on the solubility and stability of the desired component in the system.

Taken together, the kinetic study represents an essential task in assessing the extraction process as it allows estimation of the cost-effectiveness. The obtained evidence is supportive when it is necessary to choose the suitable extraction method for natural matrices, including the scale-up to an industrial level. In this case, the scaling-up of the process will be reasonable. A convenient extraction procedure, based on the use of green solvents and cheap and abundant raw material that allows one to obtain an extract with a good concentration of bioactive compounds, was proposed.

## Figures and Tables

**Table 1:** Empirical kinetic models.

Kinetic Model	Non-Linear Equation	Linearized Equation
Parabolic diffusion	$\bar{q}$ = A_0_ + A_1_t^1/2^	$\bar{q}$ = A_0_ + A_1_t^1/2^
Power law	$\bar{q}$ = Bt^n^	ln $\bar{q}$ = ln B + n ln t
Hyperbolic (Peleg’s)	$\bar{q}$ = $\frac{C_{1}t}{1+{\;C}_{2}t}$	$\frac{1}{\bar{q}}$ = $\frac{1}{C_{1}}$× $\frac{1}{t}$ + $\frac{C_{2}}{C_{1}}$
Elovich’s	$\bar{q}$ = E_0_ + E_1_ ln t	$\bar{q}$ = E_0_ + E_1_ ln t

**Table 2:** Kinetic models, constants, and regressed statistical parameters for parabolic, power law, hyperbolic, and Elovich’s models for extraction of total phenolic compound (TPC) from lentil seed coat using conventional solid–liquid extraction (CSLE) and ultrasound-assisted extraction (UAE) using different solvents.

Model	Parabolic					Power-Law					Hyperbolic					Elovich’s				
**TPC**	**R^2^**	**SEE**	**RMS**	**A_0_**	**A_1_**	**R^2^**	**SEE**	**RMS**	**B**	**n**	**R^2^**	**SEE**	**RMS**	**C_1_**	**C_2_**	**R^2^**	**SEE**	**RMS**	**E_0_**	**E_1_**
CSLE-H_2_O	0.848	0.085	4.663	5.244	0.103	0.931	0.047	4.663	5.008	0.064	0.907	0.05	4.663	18.544	3.096	0.933	0.046	4.663	4.979	0.356
CSLE-EtOH	0.77	0.139	5.383	6.07	0.077	1	0.16	5.389	3.375	0.344	0.953	0.046	5.383	27.589	4.142	0.915	0.077	5.383	5.67	0.634
CSLE-MeOH	0.766	0.389	5.888	6.179	0.174	0.909	0.174	5.389	5.751	0.344	0.903	0.154	5.887	14.703	1.959	0.916	0.077	5.888	5.67	0.634
UAE-H_2_O	0.979	0.034	1.921	2.466	1.519	0.935	0.07	1.92	3.222	0.144	0.775	0.143	1.919	51.176	15.154	0.915	0.625	1.921	3.21	0.405
UAE-EtOH	0.982	0.41	1.027	1.508	1.74	0.942	0.582	1.021	2.332	0.209	0.772	0.756	1.024	34.799	14.796	0.913	0.625	1.028	2.281	0.351
**TFC**	**R^2^**	**SEE**	**RMS**	**A_0_**	**A_1_**	**R^2^**	**SEE**	**RMS**	**B**	**n**	**R^2^**	**SEE**	**RMS**	**C_1_**	**C_2_**	**R^2^**	**SEE**	**RMS**	**E_0_**	**E_1_**
CSLE-H_2_O	0.686	0.266	4.711	2.08	0.107	0.969	0.002	1.355	2.097	0.06	0.915	0.005	1.356	8.28	3.335	0.97	0.002	1.355	2.086	0.14
CSLE-EtOH	0.951	0.049	1.581	1.871	0.174	0.98	0.02	1.58	1.618	0.237	0.916	0.085	1.575	1.982	0.605	0.969	0.03	1.58	1.475	0.575
CSLE-MeOH	0.919	0.053	1.005	1.441	0.139	0.809	0.125	1.001	1.274	0.23	0.622	0.251	0.997	1.742	0.704	0.759	0.157	1.004	1.214	0.412
UAE-H_2_O	0.746	0.012	0.788	1.923	0.365	0.857	0.008	0.799	2.116	0.057	0.832	0.007	0.807	80.589	37.211	0.86	0.008	0.8	2.116	0.118
UAE-EtOH	0.979	0.077	0.37	0.847	1.649	0.989	0.112	0.394	1.679	0.354	0.939	0.174	0.411	7.492	3.481	0.966	0.144	0.397	1.649	0.431
**CTC**	**R^2^**	**SEE**	**RMS**	**A_0_**	**A_1_**	**R^2^**	**SEE**	**RMS**	**B**	**n**	**R^2^**	**SEE**	**RMS**	**C_1_**	**C_2_**	**R^2^**	**SEE**	**RMS**	**E_0_**	**E_1_**
CSLE-H_2_O	0.961	0.013	2.316	2.903	0.101	0.939	0.02	2.316	2.721	0.102	0.787	0.071	2.316	7.111	1.968	0.927	0.024	2.316	2.69	0.326
CSLE-EtOH	0.705	0.623	3.67	3.78	0.218	0.879	0.257	3.671	3.322	0.174	0.991	0.716	3.677	55.643	11.72	0.909	0.192	3.67	3.112	0.811
CSLE-MeOH	0.923	0.141	4.851	4.905	0.232	0.953	0.086	4.851	4.484	0.137	0.847	0.282	4.849	8.756	1.324	0.944	0.102	4.851	4.374	0.769
UAE-H_2_O	0.989	0.003	2.174	2.783	1.301	0.917	0.02	2.173	3.435	0.115	0.746	0.061	2.173	68.663	19.245	0.899	0.024	2.174	3.429	0.357
UAE-EtOH	0.979	0.027	0.906	1.008	2.999	0.925	0.098	0.889	2.529	0.461	0.845	0.218	0.852	7.559	1.969	0.844	0.2	0.899	2.481	0.802

## Data Availability

The whole metabolomics dataset is provided as supplementary material.

## Supplementary Materials

The following are available online at https://www.mdpi.com/article/10.3390/foods10081810/s1, Table S1. Metabolomic dataset regarding phenolic compounds annotated in aqueous, methanolic, and ethanolic extracts obtained with CSLE and UAE using untargeted UHPLC/QTOF mass spectrometry. Compounds are grouped in phenolic sub-classes, and are provided with individual abundances, retention time, and composite mass spectra (mass-abundance combinations). Table S2. Semi-quantitative values (expressed as mg eq./Kg) for the different phenolic subclasses considering both the extraction solvent and the ultrasound assisted extraction under investigation. Table S3. Semi-quantitative values (expressed as mg eq./Kg) for the different phenolic subclasses considering the extraction solvent under investigation.
