# Supplementary material for: Extraction Kinetics of Total Polyphenols, Flavonoids, and Condensed Tannins of Lentil Seed Coat: Comparison of Solvent and Extraction Methods
Source: Foods. 2021 Aug 5;10(8):1810. doi: 10.3390/foods10081810 (PMC8393944; doi:10.3390/foods10081810)
Supplement: Supplementary file 1 [file foods-10-01810-s001.zip › foods-1313822-Table S2-3.pdf]

Table S2 Semi-quantitative values (expressed as mg eq./Kg) for the different phenolic subclasses considering both the extraction solvent and the ultrasound assisted extraction under investigation.

Extraction solvents	UAE	Anthocyanins (mg/kg)	Flavanols (mg/kg)	Flavones (mg/kg)	Flavonols (mg/kg)	Lignans (mg/kg)	Tyrosols (mg/kg)	Phenolic acids (mg/kg)	Stilbenes (mg/kg)
Water	Control	2236.34±259.61^{a}	157.28±30.58^{cde}	138.07±15.98^{ab}	53.05±11.50^{abc}	271.90±19.69^{ab}	278.37±15.65^{a}	119.59±18.13^{abc}	46.81±5.10^{ab}
	20 min	1983.73±526.01^{a}	143.67±33.04^{bcde}	144.62±27.57^{ab}	51.03±12.22^{abc}	250.80±30.83^{a}	294.18±16.09^{a}	144.80± 36.15^{bcd}	51.19±5.85^{abc}
	40 min	2196.20±532.33^{a}	147.46±27.73^{bcde}	166.85±17.00^{abc}	67.78±18.71^{bc}	279.18±37.85^{abc}	318.56±68.41^{a}	117.62± 17.24^{ab}	54.89±6.14^{abcd}
	60 min	2125.52±330.35^{a}	118.77±32.07^{ab}	135.10±19.21^{a}	70.33±13.37^{c}	267.05±31.75^{ab}	314.77±94.88^{a}	119.61± 21.13^{abc}	50.17±10.91^{abc}
	120 min	2193.92±363.43^{a}	159.59±23.68^{de}	153.35±11.20^{abc}	56.97±25.94^{abc}	292.20±17.13^{bcd}	292.77±87.45^{a}	108.05±10.10^{a}	44.00±6.65^{a}
EtOH : Water (60:40)	Control	2205.17±58.86^{a}	142.83±12.66^{bcde}	178.75±21.95^{c}	46.90±12.38^{ab}	340.46±6.52^{f}	1133.63±250.34^{b}	141.37±5.59^{bcd}	66.81±5.32d^{e}
	20 min	2139.47±451.64^{a}	122.62±9.01^{abc}	185.48±28.78^{c}	44.80±11.63^{ab}	328.19±17.59^{def}	1621.76±212.39^{c}	146.08±10.79^{cd}	68.16±8.97^{e}
	40 min	1600.13±511.13^{a}	103.42±15.20^{a}	165.31±21.51^{abc}	50.97±8.22^{abc}	302.19±28.19^{bcde}	885.86±351.23^{b}	140.61±8.12^{bcd}	57.30±6.89^{bcde}
	60 min	1600.27±325.97^{a}	127.78±5.56^{abcd}	159.58±7.24^{abc}	62.08±3.03^{abc}	332.63±17.51^{ef}	1116.40±471.83^{b}	144.12±6.13^{bcd}	60.22±12.69^{cde}
	120 min	1792.32±161.40^{a}	164.96±9.03^{e}	169.75±14.72^{bc}	42.45±8.31^{a}	315.65±1.97^{cdef}	755.58±364.67^{b}	152.59±7.43^{d}	68.52±1.04^{e}

Values with different superscripts within the same graph are significantly different for $p < 0.05$.

Table S3 Semi-quantitative values (expressed as mg eq./Kg) for the different phenolic subclasses considering the extraction solvent under investigation.

Extraction solvents	Anthocyanins (mg/kg)	Flavanols (mg/kg)	Flavones (mg/kg)	Flavonols (mg/kg)	Lignans (mg/kg)	Tyrosols (mg/kg)	Phenolic acids (mg/kg)	Stilbenes (mg/kg)
Water	1840.30±99.80^{a}	159.20±22.97^{a}	76.08±8.19^{a}	89.35±7.04^{a}	221.98±34.49^{a}	734.12±426.75^{a}	92.04±5.94^{a}	34.00±21.24^{a}
MeOH : water	2865.88±74.83^{b}	323.92±20.45^{b}	93.72±14.57^{ab}	94.65±22.13^{a}	254.77±37.28^{a}	848.53±23.68^{a}	95.30±19.91^{a}	47.28±16.02^{a}
EtOH : water	2155.77±252.17^{c}	324.77±29.14^{b}	110.69±31.46^{b}	70.59±26.90^{a}	329.45±8.94^{b}	830.71±237.08^{a}	104.62±11.55^{a}	47.87±6.06^{a}

Values with different superscripts within the same graph are significantly different for $p < 0.05$.
